# Age-associated changes in multimodal pain perception

**DOI:** 10.1093/ageing/afae107

**Published:** 2024-05-22

**Authors:** Yongkang Zhi, Yu Zhang, Yu Zhang, Ming Zhang, Yazhuo Kong

**Affiliations:** CAS Key Laboratory of Behavioral Science, Institute of Psychology, Chinese Academy of Sciences, Beijing 100101, China; Department of Psychology, University of Chinese Academy of Sciences, Beijing 100049, China; Department of Applied Psychology, School of Humanities and Social Sciences, Beijing Forestry University, Beijing 100083, China; CAS Key Laboratory of Behavioral Science, Institute of Psychology, Chinese Academy of Sciences, Beijing 100101, China; Department of Psychology, University of Chinese Academy of Sciences, Beijing 100049, China; CAS Key Laboratory of Behavioral Science, Institute of Psychology, Chinese Academy of Sciences, Beijing 100101, China; Department of Psychology, University of Chinese Academy of Sciences, Beijing 100049, China; CAS Key Laboratory of Behavioral Science, Institute of Psychology, Chinese Academy of Sciences, Beijing 100101, China; Department of Psychology, University of Chinese Academy of Sciences, Beijing 100049, China; Wellcome Centre for Integrative Neuroimaging, FMRIB, Nuffield Department of Clinical Neurosciences, University of Oxford, Oxford OX3 9DU, UK

**Keywords:** pain, somatosensory, heat, pressure, ageing, older people

## Abstract

**Background:**

Pain sensitivity varies across multimodal somatosensory stimuli that can rely on different conductive fibres, which, when damaged, will lead to neuropathies. However, there is limited research examining the characteristics of perceived pain, particularly as affected by the ageing process, as induced by various somatosensory stimuli that may rely on small or large fibres.

**Methods:**

Using heat and pressure stimuli on small and large fibres separately on both younger and older adults, this study examined age-associated changes in pain perception by measuring self-reported pain sensitivity, pain threshold and pain discriminability.

**Results:**

Heat pain threshold was significantly positively correlated with age, but not pressure pain threshold. Pain threshold increased and pain discriminability decreased in response to heat stimuli in the older participants compared with the younger ones.

**Conclusion:**

An age-associated decline in heat pain perception was observed, suggesting an earlier degradation of heat perception. These findings provide new insight into understanding and assessing somatosensory disorders, which can help ageing populations better maintain healthy sensory functioning.

## Key Points

- An age-associated decline in heat pain perception was observed in older adults.
- Older adults showed a higher heat threshold but no significant difference was seen in pressure threshold.
- Older adults showed a reduced discriminability of heat stimulation.

## Introduction

The somatosensory system, which includes the perceptions of tactile, temperature, pressure, and pain as detected through a wide range of sensory receptors [1], provides us with critical information about our internal and external environments [2]. Pain in particular is a significant alert, warning us of potential problems, damage or dangerous situations [3]. Somatosensory stimuli such as heat, cold and pressure can further arouse our perception of physical pain as their intensity increases [4].

Ageing is a natural process during which human experience declines in their somatosensory systems [5, 6], and a change in pain experience is one typical effect of ageing [7, 8]. Somatosensory declines tend to make individuals more susceptible to injury [2], suggesting age-related decreases in pain sensitivity [9]. Previous studies have shown a sensory threshold increase in older adults [10]. However, few studies have distinguished between the degeneration processes of the different somatosensory types. Furthermore, findings regarding age-related changes in pain perception have been inconsistent due to the use of differing pain induction methods [11], as pain sensitivities vary according to stimulation type (e.g. increasing pressure pain versus unchanged heat pain) [11].

Changes in pain perception due to ageing could be related to different somatosensory sensations. Multimodal somatosensation relies on different types of conductive fibres [12]. Peripheral nerve fibres contain both small and large fibres, both of which detect sensations in the skin and carry their respective signals to the central nervous system [13]. Small fibres (e.g. C, Aδ fibres) respond mainly to external thermal, mechanical, or chemical stimulations [14], while tactile and pressure sensations on the skin are often related to large fibres (e.g. Aβ fibre) [15]. Damage to the fibres can cause neuropathies characterised by positive or negative symptoms related to sensation (e.g. neuropathic pain [16], sensory loss [17–21]). Thermal stimulation and pressure can activate small (Aδ and C) and large (Aβ) fibres separately [15, 22–24]; therefore, these stimuli are often used to detect fibre damage [25].

The effects of age-related changes in pain perception are complicated. Pain is a subjective experience, and a complex sensation [26]. Multiple cognitive and emotional factors are known to modulate an individual’s pain experience [27]. The prevalence of chronic pain is known to increase as ageing advances [26], which means that older adults tend to experience more pain-related issues [28]. Furthermore, past painful experiences can also influence older people’s tolerance of painful situations [28].

Considering the potential associations between ageing and pain experience [29], this study aimed to measure age-associated changes in pain perception, in both small and large fibres, using somatosensory rating (i.e. warmth, pressure), pain threshold (i.e. pain from heat, pain from pressure) and pain discriminability (i.e. high versus low intensity). To invoke the sensation of pain, heat and pressure stimuli were administered at various intensities to both older and younger adults. The intent was to obtain measurements from which to develop consultation guidelines when assessing someone for somatosensory disorders or predicting treatment outcomes.

## Methods

### Participants

A priori power analysis demonstrated that a sample size of 12 would allow for detecting an effect size (*f* = 0.25) with 80% power at an *α* of 0.05 for the repeated measures with one between-participant (i.e. group) and two within-participant variables (i.e. stimulation and intensity) using G^*^Power Version 3.1, according to the guide proposed by Bartlett (2022) (https://osf.io/zqphw/). Thirty-two healthy young adults recruited from the local universities (16 male, age = 23.63 ± 2.37 years, range = 19–28 years) participated in the younger group, and 30 healthy middle-aged or older adults recruited from the local communities (15 male, age = 54.70 ± 9.70 years, range = 40–72 years) in the older group took part in this study. All research procedures were approved by the local institutional review board. All participants in the current research were instructed to not ingest any alcohol or pain medicine for at least four hours before participating in the experiment. After arriving at the lab, each participant underwent a thorough written and verbal informed consent process. Participants were fully debriefed and compensated for their participation after completing all tasks.

### Stimuli

#### Heat stimulation

The heat pain stimuli were produced using a Medoc 9-cm^2^ Contact Heat-Evoked Potential Stimulator (Medoc Ltd, Ramat Yishai, Israel). The heat pain threshold (younger adults: 43.02 ± 2.73°C; older adults: 45.72 ± 2.07°C) was conducted first to determine whether the designed stimuli would induce pain sensations of different intensities. The heat pain threshold was assessed by administering the probe five times on the participant’s right foot, 3 cm above the middle toe, with a temperature rise rate of 0.5°C/s. Participants indicated when they first began to feel pain by pressing a button that turned the device off. During the main experiment, 5 s heat stimuli with a temperature rise rate of 40°C/s were applied. Forty-five heat pulses measured participants’ perception of the heat stimuli pain at nine intensity levels: 39°C, 40°C, 41°C, 42°C, 43°C, 44°C, 45°C, 46°C and 47°C for younger adults, and 42°C, 43°C, 44°C, 45°C, 46°C, 47°C, 48°C, 49°C and 50°C for older adults. The inconsistent intensity settings are due to the fact that most younger adults were intolerant of the higher thermal stimulation (i.e. 49°C and above), and some older adults failed to detect their heat perception by lower thermal stimulation (i.e. 41°C and below) or were unable to detect their heat pain threshold using the highest intensity administered to the younger group (i.e. 47°C) in our pilot testing experiment. Participants were asked to report the pain they felt during the brief heat stimuli using a numerical pain rating scale ranging from 0 to 10 (0 = no feeling, 1 = a feeling of warmth, 2 = a feeling of heat, 3 = a feeling of hotness, 4 = beginning to feel pain, 10 = a feeling of pain as bad as it could be) [30]. Values from 4 to 10 gradually increased as the degree of pain also increased.

#### Pressure stimulation

The pressure stimuli were produced using a MRI-Compatible Foot-Sole Stimulation System [31]. The pressure pain threshold (younger adults: 164.36 ± 45.57 N; older adults: 160.32 ± 35.13 N) was first assessed to determine whether the designed stimuli would induce the pain sensations at different intensities. The pressure pain threshold was determined by administering the probe five times to the sole of the participant’s left foot, 3 cm below the middle toe, with a pressure increase of 12 N/s. Participants indicated the point when they began to feel pain through oral report. Next, 5 s of pressure stimuli were then applied in the main experiment. Participants’ pain perception of the pressure stimuli was measured by participants’ responses to 45 pressure pulses at intensities of 100 N, 120 N, 140N, 160 N, 170 N, 180 N, 210 N, 220 N and 250 N in both groups. Participants were asked to report the pain they experienced during the brief pressure stimuli using a numerical pain rating scale ranging from 0 to 10 (0 = no feeling, 1 = a feeling of being touched, 2 = a moderate feeling of pressure, 3 = a strong feeling of pressure, 4 = beginning to feel pain, 10 = a feeling of pain as bad as it could be). Values from 4 to 10 gradually increased along with the degree of pain.

### Procedure

**Figure 1:**
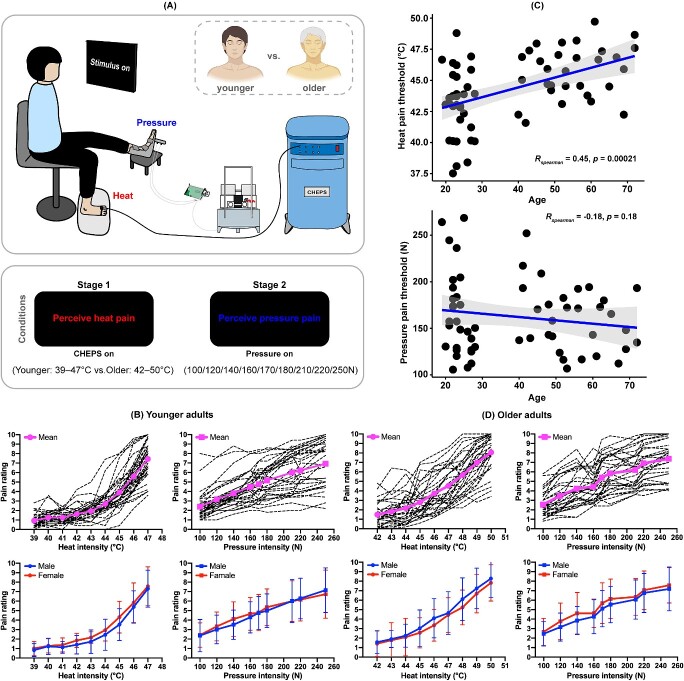
Pain perception elicited by multiple somatosensory stimuli. (A) Experimental setup and conditions (created with BioRender.com). (B) Pain rating of younger adults regarding heat and pressure stimuli at various intensities. (C) Correlation between age and pain thresholds. (D) Older adults’ pain ratings for heat and pressure stimuli at various intensities.

Upon arriving at the lab, and after providing their informed consent, participants were asked to respond to the Pain Sensitivity Scale (PSQ, e.g. ‘Imagine you burn your tongue on a very hot drink,’ responses rated on a 10-point scale ranging from 1 = no pain to 10 = pain as bad as it could be [32]). Then, the participants completed the two-stage main experiment (Figure 1A). The heat and pressure stimuli were applied separately to participants’ right and left feet. Stage 1 measured participants’ perception of one type of stimuli. After assessing the pain threshold, the pain ratings of the heat stimuli were measured according to participants’ responses to 45 heat pulses at various intensities. The presentation of stimuli and manual response measurements were controlled using E-Prime 3.0 (Psychological Software Tools, Inc., Pittsburgh, PA, USA). In each trial, a white fixation cross was first presented for 1 s, followed by instructions (i.e. ‘Heat stimulation on’), shown for 5 s. Meanwhile, a heat pulse was delivered to the right foot. Instructions were then shown on the screen for 5 s, asking participants to consider the sensation they experienced and report a pain rating for the stimuli using the numerical pain rating scale using a response box located by their right hand. After this, a black background screen appeared for 10 s before the subsequent trial began. Stage 2 measured participants’ perception of the other pain stimulus. After assessing the pressure pain threshold, the pain ratings of the pressure stimuli were also measured using participants’ responses to 45 pressure pulses given at various intensities. The setup of each trial was similar to that in Stage 1 but with the instructions written to reflect the given context (i.e. ‘Pressure stimulation on’). The order of the two measured stimuli stages was counterbalanced across all participants.

## Results

### Perceptions of heat and pressure stimuli

We first calculated the mean ratings of the heat and pressure stimuli at each level of intensity (Table 1). We expected that the higher the stimulus intensity, the higher the score (Figure 1B and D). We found that the heat threshold (but not pressure threshold) was positively significantly correlated with age across the two groups (*r_heat-threshold_* = 0.45, *P* < 0.001, Figure 1C; *r_pressure-threshold_* = −0.175, *P* = 0.18), suggesting that heat threshold increases with age. Moreover, younger adults’ heat pain threshold was significantly positively correlated with their pressure pain threshold (*r* = 0.475, *P* = 0.009), but there was no similar significant correlation in the older adults group (*r* = −0.173, *P* = 0.36). In both the younger and older adult groups, no significant gender difference was seen in the pain ratings for heat or pressure stimuli at all levels of intensity (*ps_younger_* ≥ 0.154, *ps_older_* ≥ 0.278).

**Table 1:** Mean ratings for heat and pressure stimuli at each intensity level

**Heat intensity**	***M* ± *SD***		**Pressure intensity**	***M* ± *SD***	
	**Younger**	**Older**		**Younger**	**Older**
39°C	0.93 ± 0.71	—	100N	2.39 ± 1.48	2.55 ± 1.34
40°C	1.26 ± 0.79	—	120N	3.17 ± 1.50	3.46 ± 1.71
41°C	1.25 ± 0.69	—	140N	3.81 ± 1.68	4.24 ± 1.88
42°C	1.63 ± 0.89	1.49± 1.26	160N	4.47 ± 1.67	4.44± 1.64
43°C	1.94 ± 0.99	1.83 ± 1.44	170N	4.77 ± 1.80	5.40 ± 2.01
44°C	2.69 ± 1.41	2.18 ± 1.61	180N	5.17 ± 1.84	5.84 ± 1.99
45°C	3.92 ± 1.69	2.79 ± 1.76	210N	6.00 ± 2.17	6.21 ± 1.98
46°C	5.61 ± 1.90	3.72 ± 1.89	220N	6.21 ± 2.16	6.91 ± 2.01
47°C	7.44 ± 1.97	4.54 ± 2.02	250N	6.94 ± 2.42	7.38 ± 2.07
48°C	—	5.67 ± 2.06			
49°C	—	6.97 ± 2.07			
50°C	—	8.05 ± 1.93			

### Heat perception degeneration in older adults

**Figure 2:**
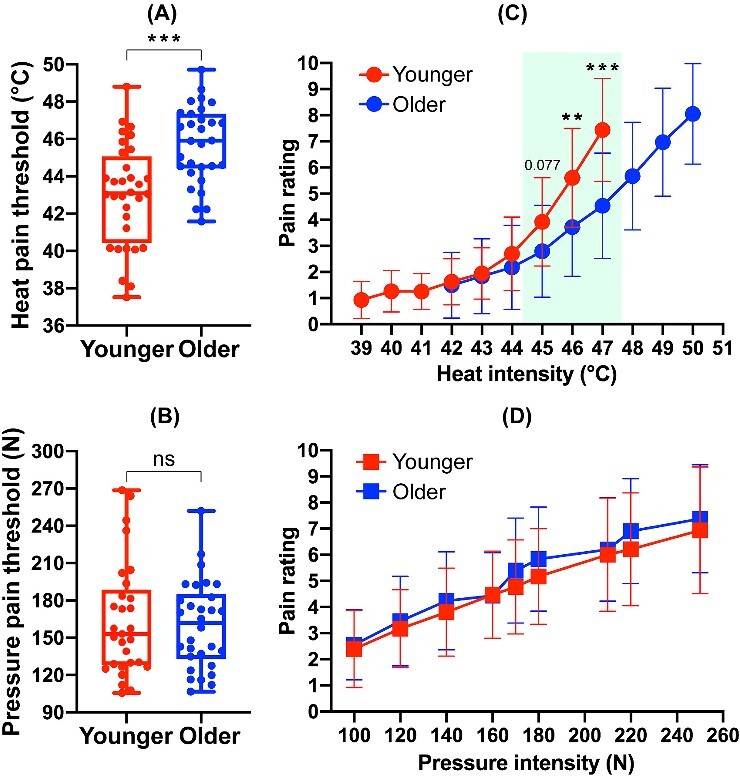
Comparison of pain perception between younger and older adults. (A) Significant difference in heat threshold. (B) Non-significant difference in pressure threshold. (C) Pain ratings of heat stimuli across younger and older groups. (D) Pain ratings of pressure stimuli across younger and older groups (^*^^*^P < 0.01, ^*^^*^^*^P< 0.001).

To explore the degeneration of pain perception of different types of noxious stimuli, we compared all the pain assessment dimensions between the younger and older adult groups. Compared to younger adults, older adults reported a higher heat threshold (younger adults: 43.02 ± 2.73°C, older adults: 45.72 ± 2.07°C, *t*(60) = 4.36, *p_heat-threshold_* < 0.001, *d* = 1.11; Figure 2A). No substantial difference was seen between the two groups’ pressure thresholds (younger adults: 164.36 ± 45.57 N, older adults: 160.32 ± 35.13 N, *t*(60) = −0.38, *p_pressure-threshold_* = 0.704; Figure 2B). This implies a degeneration of heat pain perception as we age. Meanwhile, the pain ratings of heat stimuli with supra-threshold intensities for the older group was significantly lower than those for the younger group (younger adults: 3.92 ± 1.69, older adults: 2.79 ± 1.76*, t*(60) = −2.57, *p_45°C_* = 0.013, *p_Bonferroni-corrected_* = 0. 0.0771 (R Studio 2022.07.1), *d* = 0.65 for 45°C; younger adults: 5.61 ± 1.90, older adults: 3.72 ± 1.89, *t*(60) = −3.93, *p_46°C_* < 0.001, *p_Bonferroni-corrected_* = 0.0014, *d* = 1.00 for 46°C; younger adults: 7.44 ± 1.97, older adults: 4.54 ± 2.02*, t*(60) = −5.72, *p_47°C_* < 0.001, *p_Bonferroni-corrected_* = 0.000006, *d* = 1.45 for 47°C; Figure 2C). There was no significant difference seen in the ratings of pressure stimuli (*ps* ≥ 0.173, Figure 2D), and the self-reported pain sensitivity between the younger and older adult groups (i.e. PSQ score; younger adults: 4.59 ± 1.37, older adults: 5.20 ± 1.72, *t*_(60)_ = 1.54, *p_pain-sensitivity_* = 0.128).

### Pain discriminability discrepancy

**Figure 3:**
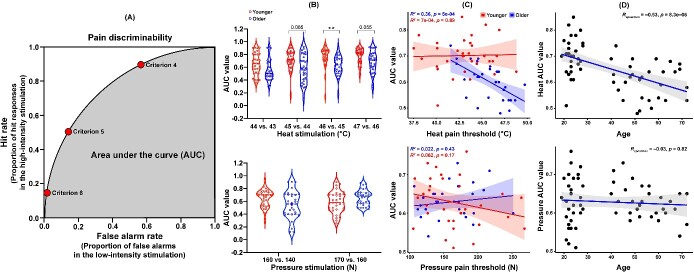
Pain discriminability by AUC values across younger and older groups. (A) Schematic diagram describing the calculation of the discriminability index (modified from [34]). (B) Significant difference in pain discriminability at various levels of heat and pressure stimuli. (C) Correlations between pain discriminability and pain threshold. (D) Correlations between age and discriminability of heat stimulation (^*^^*^P < 0.01).

As impaired somatosensory discriminability (i.e. tactile discrimination) has often been observed in patients experiencing chronic pain [33], we applied an approach to address pain discriminability (i.e. qualified by the area under the curve [AUC] value, which is an oft-used nonparametric measure of discriminability) as proposed by Zhang et al. (2022) to quantify sensory discriminability between the younger and older adult groups [34]. This approach used a rating design in the framework of Signal Detection Theory to calculate the participant’s ability to distinguish between high-intensity stimulation (i.e. signal) and low-intensity stimulation (i.e. noise). Here, participants report pain by a numerical rating scale. This approach was equivalent to treating every numerical rating (0–10) on the scale as an implicit criterion [34]. To calculate the AUC values, for a given criterion (e.g. 6 on the numerical pain rating scale), ratings greater than or equal to this criterion (i.e. 6–10) in the high-intensity stimulation were hit responses, whereas ratings greater than or equal to this criterion in the low-intensity stimulation were false alarms. The hit rate was obtained by dividing the number of hit responses by the number of total trials in the high-intensity stimulation, and the false alarm rate was defined as the proportion of false alarms in the low-intensity stimulation. A hit rate-false alarm rate pair defined a point on the ROC curve (Figure 3A). The numerical rating scale had 11 integral ratings, so there would be 11 points on the ROC curve, and the AUC value was defined as the area under the ROC curve [34]. We calculated the AUC values for each pair of low-high intensity stimuli sequentially (e.g. 45°C versus 46°C for heat stimulation, 160 N vs. 170 N for pressure stimulation), and compared the AUC values of the pain discrimination index between the younger and older adult groups (Table 2). We found that the mean discriminability of heat stimulation (but not pressure stimulation) was significantly negatively correlated with age among both groups (*r_heat_* = −0.53, *p* < 0.001; *r_pressure_* = −0.03, *P* = 0.82, Figure 3D), suggesting that the discriminability of heat stimulation declines as we age.

**Table 2:** Comparison of pain discriminability between younger and older adults

**Comparison**	***M* ± *SD***		***t***	***P***	***p***__***Bonferroni-corrected***__
	**Younger**	**Older**			
**Heat**					
AUC_43vs42_	0.59 ± 0.14	0.57 ± 0.15	0.52	0.607	—
AUC_44vs43_	0.66 ± 0.17	0.55 ± 0.17	2.39	**0.02**	0.1
AUC_45vs44_	0.72 ± 0.16	0.60 ± 0.22	2.56	**0.013**	0.065
AUC_46vs45_	0.77 ± 0.16	0.63 ± 0.15	3.55	**0.001**	**0.005**
AUC_47vs46_	0.78 ± 0.12	0.68 ± 0.17	2.64	**0.011**	**0.055**
**Pressure**					
AUC_120vs100_	0.71 ± 0.14	0.73 ± 0.12	−0.54	0.589	—
AUC_140vs120_	0.68 ± 0.13	0.67 ± 0.14	0.19	0.852	—
AUC_160vs140_	0.65 ± 0.13	0.55 ± 0.22	2.26	**0.029**	0.232
AUC_170vs160_	0.59 ± 0.17	0.66 ± 0.12	−2.03	**0.047**	0.376
AUC_180vs170_	0.54 ± 0.14	0.56 ± 0.10	−0.74	0.461	—
AUC_210vs180_	0.68 ± 0.16	0.61 ± 0.16	1.62	0.11	—
AUC_220vs210_	0.58 ± 0.15	0.61 ± 0.16	−0.69	0.496	—
AUC_250vs220_	0.61 ± 0.16	0.65 ± 0.17	−1.01	0.316	—

Note: The bolded value means reaching a significant level.

We found that the AUC values of the older adult group were significantly lower than those of the younger adult group, especially for stimuli with intensity around or above the participants’ pain threshold (e.g. 45°C versus 44°C, 46°C versus 45°C and 47°C versus 46°C; Table 2, Figure 3B), also suggesting a decline in the discriminability of heat stimulation as we age. Meanwhile, the older group’s discriminability of heat stimuli was significantly negatively correlated to their heat pain threshold (*r_older_* = −0.60, *P* < 0.001; *r_younger_* = 0.03, *P* = 0.89, Figure 3C), meaning that the higher one’s pain threshold, the worse their discriminability, verifying a potential deterioration of the perception of heat stimulation.

We also observed some unstable changes in the discriminability of pressure stimuli when comparing the intensities around the pressure threshold. The older group showed decreased discriminability in the contrast of 160 N versus 140 N (below their pressure pain threshold), but increased discriminability in the contrast of 170 N vs. 160 N (above their pressure threshold), compared with the younger group (Table 2, Figure 3B); however, *P* values could not survive multiple comparison corrections. It suggests that the discriminability of pressure may remain relatively stable as we age. However, the discriminability of pressure stimulation was not significantly correlated with participants’ pressure threshold, *r_older_* = 0.15, *P* = 0.433; *r_younger_* = −0.28, *P* = 0.148.

## Discussion

This study compared pain perception as induced by different somatosensory modalities in both older and younger adults using pressure and heat stimuli with various intensities. We observed an age-associated decline in heat pain perception. Older adults showed decreased pain sensitivity to heat stimuli, that is, a higher heat threshold, but no significant change in pressure threshold. Older adults also demonstrated a reduced discriminability of heat stimuli compared to younger adults. These findings provide insight into somatosensory degradation and its assessment in older adults, potentially contributing to further individualised rehabilitation therapy.

This study shows that the heat pain threshold in older adults increases compared with that of younger adults, suggesting a degradation of heat pain sensitivity. We found that age appears to have a stronger effect on heat sensation (conducted by Aδ and C fibres) compared with pressure sensation (conducted by Aβ fibres). This threshold can yield immediate information as an indicator of somatosensory functioning [35–37].

Age-related differences in pain sensitivity vary across different stimulus modalities [11]. Heat threshold deviations may be an early indicator of ageing. Pain threshold depends on both noxious peripheral input from superficial tissue and the modulatory processing of the central nervous system [38, 39]. At the peripheral level, multiple stimuli-induced somatosensations activate different fibres in the body, which can explain variations in pain thresholds for different sensations. In the current study, the heat stimulus was conducted by small fibres (i.e. Aδ and C fibres) [22], whereas the pressure stimulus was conducted by large fibres (i.e. Aβ fibres) [15]. Both heat and pressure stimuli activate touch-sensitive afferents, however, heat stimuli activate skin nociception, while pressure stimuli target both skin and deep tissue nociceptors [40]. Age-related decrease in heat pain perception may be related to one’s degeneration of the degree of skin innervation with reference to peripheral small fibres [24]. The observed correlation of pain threshold between heat and pressure in younger adults, but not in older adults, also suggests heat-specific changes in older adults. At the central level, age-related differences in phase synchrony between prefrontal regions and sensory cortices suggested one possibility for accounting for deficits in sensory inhibition in older healthy adults [41]. Meanwhile, increased prevalence or severity of disease in older adults could also be associated with age-related changes in central pain sensitivity [26].

Decreased discrimination of heat stimuli, which appeared to occur in parallel with a pain reduction in our study, further confirms the degradation phenomenon of heat pain perception in older adults. The ability to distinguish between stimuli of different intensities is a predominant function of pain perception, indicating the nervous system’s selective encoding of relevant information [34]. For those suffering from chronic pain, it is beneficial to be able to distinguish between painful and non-painful somatosensations, and different sensory qualities of pain [42]. Heat pain is a complicated sensation, comprising the initial sense of pain, which is sharp and very localised based on Aδ fibres, and then followed by secondary pain, a diffuse burning sensation based on C fibres [43]. Reduced discriminability of heat stimulation may result from reorganisation at the somatosensory cortex level due to peripheral and central ageing [44]. Indeed, impaired perceptual discrimination is often observed in older adults suffering from chronic pain [42].

One limitation of the current study is that the age distribution in older adults group was somewhat discrete, while the age distribution in younger adults group was quite concentrated. The recruitment range in this study was from 40 to 75 years of age, with one male and one female recruited for each 3-year age interval. The older group did in fact include participants who were in middle-adulthood as well as seniors, which does not allow for the generalisation of our results to very old individuals. Nevertheless, our results reveal an apparent ageing-related characteristic of diminishing pain perception of heat stimuli. We recommend that future studies further characterise pain perception using additional somatosensory modalities, and using samples that include more divisions in age groups and larger sample sizes. The second limitation is that the same step did not regularly increase the intensity levels of pressure stimuli due to adjusting stimulation settings to distinguish all measures. Future research should use more appropriate stimulation ranges and intensities to measure pressure perception in more detail.

These findings advance our understanding of age-associated declines in sensorimotor functioning and contribute to better assessing the sensory function of patients with somatosensory disorders (e.g. strokes) as they age. Most importantly, it will benefit consideration when developing and selecting optimal, age-appropriate treatment strategies to decrease patients’ pain, particularly in avoiding improper adoptions of physical therapy (e.g. heat or cold packs, massage).

## References

[1] Riera CE , Dillin A. Emerging role of sensory perception in aging and metabolism. Trends Endocrinol Metab 2016; 27: 294–303. 27067041 10.1016/j.tem.2016.03.007

[2] Heft M , Robinson M. Somatosensory function in old age. J Oral Rehabil 2017; 44: 327–32. 28130938 10.1111/joor.12488

[3] Chen Y-C , Auer-Grumbach M, Matsukawa S et al. Transcriptional regulator PRDM12 is essential for human pain perception. Nat Genet 2015; 47: 803–8. 26005867 10.1038/ng.3308 PMC7212047

[4] Gruss S , Geiger M, Werner P et al. Multi-modal signals for analyzing pain responses to thermal and electrical stimuli. J Vis Exp 2019; 146: e59057. 10.3791/59057 31009005

[5] Ajayi AF , Onaolapo MC, Omole AI, Adeyemi WJ, Oluwole DT. Mechanism associated with changes in male reproductive functions during ageing process. Exp Gerontol 2023; 179: 112232. 37315721 10.1016/j.exger.2023.112232

[6] Gorniak SL , Ochoa N, Cox LIG et al. Sex-based differences and aging in tactile function loss in persons with type 2 diabetes. PloS One 2020; 15: e0242199. 33180801 10.1371/journal.pone.0242199 PMC7660517

[7] Johnson AJ , Wilson AT, Laffitte Nodarse C et al. Age differences in multimodal quantitative sensory testing and associations with brain volume. Innov Aging 2021; 5: igab033. 34616958 10.1093/geroni/igab033 PMC8489433

[8] Yıldırım E , Güntekin B, Hanoğlu L, Algun C. EEG alpha activity increased in response to transcutaneous electrical nervous stimulation in young healthy subjects but not in the healthy elderly. PeerJ 2020; 8: e8330. 31938578 10.7717/peerj.8330 PMC6953335

[9] Lautenbacher S. Experimental approaches in the study of pain in the elderly. Pain Med 2012;13:S44–50. 10.1111/j.1526-4637.2012.01326.x 22497747

[10] Heft M, Robinson M. Age differences in orofacial sensory thresholds. J Dent Res 2010;89:1102–5. 10.1177/0022034510375287 PMC3318051 20651093

[11] Lautenbacher S , Kunz M, Strate P, Nielsen J, Arendt-Nielsen L. Age effects on pain thresholds, temporal summation and spatial summation of heat and pressure pain. Pain 2005; 115: 410–8. 15876494 10.1016/j.pain.2005.03.025

[12] Valeriani M , Mazzone P, Restuccia D, Insola A. Contribution of different somatosensory afferent input to subcortical somatosensory evoked potentials in humans. J Neurol Sci 2021; 429: 118536. 10.1016/j.clinph.2021.06.033 34454262

[13] Sène D . Small fiber neuropathy: diagnosis, causes, and treatment. Joint Bone Spine 2018; 85: 553–9. 29154979 10.1016/j.jbspin.2017.11.002

[14] Finsterer J , Scorza FA. Small fiber neuropathy. Acta Neurol Scand 2022; 145: 493–503. 35130356 10.1111/ane.13591

[15] Ge Y , Ye S, Zhu K et al. Mediating different-diameter Aβ nerve fibers using a biomimetic 3D TENS computational model. J Neurosci Methods 2020; 346: 108891. 32798529 10.1016/j.jneumeth.2020.108891

[16] Ye Q , Huang Z, Lu W et al. Identification of the common differentially expressed genes and pathogenesis between neuropathic pain and aging. Front Neurosci 2022; 16: 994575. 36340779 10.3389/fnins.2022.994575 PMC9626798

[17] Itani M , Gylfadottir SS, Krøigård T et al. Small and large fiber sensory polyneuropathy in type 2 diabetes: influence of diagnostic criteria on neuropathy subtypes. J Peripher Nerv Syst 2021; 26: 55–65. 33295647 10.1111/jns.12424

[18] Raasing LR , Vogels OJ, Veltkamp M, van Swol CFP, Grutters JC. Current view of diagnosing small fiber neuropathy. J Neuromuscul Dis 2021; 8: 185–207. 33337383 10.3233/JND-200490 PMC8075405

[19] Hulens M , Bruyninckx F, Thal DR, Rasschaert R, Bervoets C, Dankaerts W. Large-and small-fiber neuropathy in patients with Tarlov cysts. J Pain Res 2022; 15: 193–202. 35115823 10.2147/JPR.S342759 PMC8801331

[20] Rasmussen VF , Jensen TS, Tankisi H et al. Large fibre, small fibre and autonomic neuropathy in adolescents with type 1 diabetes: a systematic review. J Diabetes Complications 2021; 35: 108027. 34429229 10.1016/j.jdiacomp.2021.108027

[21] Morgalla MH , Domay L. Analysis of somatosensory profiles using quantitative sensory testing during tonic and BurstDR stimulation for the treatment of chronic pain. Pain Physician 2022; 25: 373–80. 35901477

[22] Van Neerven SG , Mouraux A. Capsaicin-induced skin desensitization differentially affects a-delta and c-fiber-mediated heat sensitivity. Front Pharmacol 2020; 11: 615. 32508630 10.3389/fphar.2020.00615 PMC7248294

[23] Nemenov MI , Singleton JR, Premkumar LS. Role of mechanoinsensitive nociceptors in painful diabetic peripheral neuropathy. Curr Diabetes Rev 2022; 18: 97–112. 10.2174/1573399818666211208101555 34879806

[24] Wu S-W , Wang Y-C, Hsieh P-C et al. Biomarkers of neuropathic pain in skin nerve degeneration neuropathy: contact heat-evoked potentials as a physiological signature. Pain 2017; 158: 516–25. 28129214 10.1097/j.pain.0000000000000791

[25] Sharma S , Vas P, Rayman G. Small fiber neuropathy in diabetes polyneuropathy: is it time to change? J Diabetes Sci Technol 2022; 16: 321–31. 33840265 10.1177/1932296821996434 PMC8861803

[26] McGlone F , Reilly D. The cutaneous sensory system. Neurosci Biobehav Rev 2010; 34: 148–59. 19712693 10.1016/j.neubiorev.2009.08.004

[27] Terry EL , Tanner JJ, Cardoso JS et al. Associations between pain catastrophizing and resting-state functional brain connectivity: ethnic/race group differences in persons with chronic knee pain. J Neurosci Res 2022; 100: 1047–62. 35187703 10.1002/jnr.25018 PMC8940639

[28] Cole LJ , Farrell MJ, Gibson SJ, Egan GF. Age-related differences in pain sensitivity and regional brain activity evoked by noxious pressure. Neurobiol Aging 2010; 31: 494–503. 18513833 10.1016/j.neurobiolaging.2008.04.012

[29] González-Roldán AM , Terrasa JL, Sitges C, van der Meulen M, Anton F, Montoya P. Age-related changes in pain perception are associated with altered functional connectivity during resting state. Front Aging Neurosci 2020; 12: 116. 32457594 10.3389/fnagi.2020.00116 PMC7221150

[30] Zhang M , Lin X, Zhi Y, Mu Y, Kong Y. The dual facilitatory and inhibitory effects of social pain on physical pain perception. Iscience 2024; 27: 108951. 38323007 10.1016/j.isci.2024.108951 PMC10844037

[31] Zhang T , Zhang K, Zhou J et al. An MRI-compatible foot-sole stimulation system enabling characterization of the brain response to walking-related tactile stimuli. Front Neurosci 2019; 13: 1075. 31680815 10.3389/fnins.2019.01075 PMC6811610

[32] Quan X , Fong DYT, Leung AYM, Liao Q, Ruscheweyh R, Chau PH. Validation of the mandarin Chinese version of the pain sensitivity questionnaire. Pain Pract 2018; 18: 180–93. 28422444 10.1111/papr.12587

[33] Pleger B , Ragert P, Schwenkreis P et al. Patterns of cortical reorganization parallel impaired tactile discrimination and pain intensity in complex regional pain syndrome. Neuroimage 2006; 32: 503–10. 16753306 10.1016/j.neuroimage.2006.03.045

[34] Zhang L-B , Lu X-J, Huang G et al. Selective and replicable neuroimaging-based indicators of pain discriminability. Cell Rep Med 2022; 3: 100846. 36473465 10.1016/j.xcrm.2022.100846 PMC9798031

[35] Harju E-L . Cold and warmth perception mapped for age, gender, and body area. Somatosens Mot Res 2002; 19: 61–75. 11962648 10.1080/08990220120113057

[36] Ruitenberg MF , Cassady KE, Reuter-Lorenz PA, Tommerdahl M, Seidler RD. Age-related reductions in tactile and motor inhibitory function start early but are independent. Front Aging Neurosci 2019; 11: 193. 31417396 10.3389/fnagi.2019.00193 PMC6682653

[37] Tseng M-T , Chiang M-C, Yazhuo K, Chao CC, Tseng WYI, Hsieh ST. Effect of aging on the cerebral processing of thermal pain in the human brain. Pain 2013; 154: 2120–9. 23811039 10.1016/j.pain.2013.06.041

[38] El Tumi H , Johnson M, Dantas P et al. Age-related changes in pain sensitivity in healthy humans: a systematic review with meta-analysis. Eur J Pain 2017; 21: 955–64. 28230292 10.1002/ejp.1011

[39] García-Piqueras J , García-Mesa Y, Cárcaba L et al. Ageing of the somatosensory system at the periphery: age-related changes in cutaneous mechanoreceptors. J Anat 2019; 234: 839–52. 30924930 10.1111/joa.12983 PMC6539748

[40] Kosek E , Ekholm J, Hansson P. Pressure pain thresholds in different tissues in one body region. The influence of skin sensitivity in pressure algometry. Scand J Rehabil Med 1999; 31: 89–93. 10380724 10.1080/003655099444597

[41] Alain C , Chow R, Lu J et al. Aging enhances neural activity in auditory, visual, and somatosensory cortices: the common cause revisited. J Neurosci 2022; 42: 264–75. 34772740 10.1523/JNEUROSCI.0864-21.2021 PMC8802933

[42] Zaman J , Vlaeyen JW, Van Oudenhove L et al. Associative fear learning and perceptual discrimination: a perceptual pathway in the development of chronic pain. Neurosci Biobehav Rev 2015; 51: 118–25. 25603316 10.1016/j.neubiorev.2015.01.009

[43] Defrin R , Givon R, Raz N, Urca G. Spatial summation and spatial discrimination of pain sensation. Pain 2006; 126: 123–31. 16860477 10.1016/j.pain.2006.06.026

[44] Pleger B , Tegenthoff M, Ragert P et al. Sensorimotor returning in complex regional pain syndrome parallels pain reduction. Ann Neurol 2005; 57: 425–9. 15732114 10.1002/ana.20394

